# Change in Paralytic Shellfish Toxins in the Mussel *Mytilus galloprovincialis* Depending on Dynamics of Harmful *Alexandrium catenella* (Group I) in the Geoje Coast (South Korea) during Bloom Season

**DOI:** 10.3390/toxins12070442

**Published:** 2020-07-07

**Authors:** Seung Ho Baek, Jung Min Choi, Minji Lee, Bum Soo Park, Yuchengmin Zhang, Osamu Arakawa, Tomohiro Takatani, Joong-Kyun Jeon, Young Ok Kim

**Affiliations:** 1 Risk Assessment Research Center, KIOST (Korea Institute of Ocean Science and Technology), Geoje 53201, Korea; baeksh@kiost.ac.kr (S.H.B.); mjlee@kiost.ac.kr (M.L.); 2 Marine Ecosystem Research Center, KIOST, Busan 49111, Korea; jmchoi@kiost.ac.kr (J.M.C.); parkbs@kiost.ac.kr (B.S.P.); 3 Graduate School of Fisheries and Environmental Sciences, Nagasaki University, Nagasaki 852-8521, Japan; laozhangyu1993@gmail.com (Y.Z.); arakawa@nagasaki-u.ac.jp (O.A.); taka@nagasaki-u.ac.jp (T.T.); 4 Faculty of Marine Bioscience and Technology, Gangneung-Wonju National University, Gangneung 26403, Korea; jkjeon@gwnu.ac.kr; 5 Marine Environmental & Climate Research Division, KIOST, Busan 49111, Korea

**Keywords:** dinoflagellate, *Alexandrium catenella*, massive blooms, mussels *Mytilus galloprovincialis*, paralytic shellfish toxin, Geoje coast (South Korea)

## Abstract

Paralytic shellfish toxins (PSTs) produced by *Alexandrium* *catenella* (formerly *A. tamarense*) in Korean coastal waters caused the deaths of four people (in 1986 and 1996) who consumed contaminated mussels (*Mytilus edulis*). This led to more detailed consideration of the risks of PST outbreaks and incidents in Korea, including the introduction of shellfish collection bans. In this study, we investigated the relationships between *A. catenella* population dynamics and PST accumulation in the mussel *M. galloprovincialis.* Discharges from the Nakdong River affect the environmental conditions along the Geoje coast, resulting in low salinity and high nutrient levels that trigger blooms of *A. catenella*. At the toxin peak on 24 April 2017, the toxins detected in *A. catenella* cells were C1, gonyautoxin (GTX)1 and GTX2, whereas the concentrations of PSTs in *M. galloprovincialis* were high and in the order of GTX4 > GTX1 > GTX3 > saxitoxin (STX) > GTX2 > neoSTX > decarbamoylgonyautoxin (dcGTX)2 > dc GTX3. The PST level in mussels was also high. At 15 °C, the PSTs are constantly found to be higher (10-fold higher in 2017 and 30-fold higher in 2018) than safe levels for human consumption (80 μg STX diHCl equivalents 100 g^−1^).

## 1. Introduction

Harmful algal blooms (HABs) caused by *Alexandrium* species are a growing environmental problem globally and have negative impacts on marine resources and human health [1]. Members of this genus produce paralytic shellfish toxins (PSTs) [2], which accumulate in filter feeding bivalves and cause illness and death, i.e., paralytic shellfish poisoning (PSP) in human consumers [3,4]. It is important to have a solid understanding of the factors that influence the biosynthesis and accumulation of PSTs because toxic dinoflagellates are an ongoing problem for the seafood industry and continue to threaten human health worldwide [3,5,6]. In Korea, the deaths of four people in 1986 and 1996 were associated with PSTs caused by consumption of the mussel *Mytilus galloprovincialis* (formerly *M. edulis*) in the Geoje and Busan areas [7,8]. HABs associated with PSTs in Korea have mainly been attributed to the species *Alexandrium catenella* (formerly *A. tamarense*), which has been restricted to the southeast coast, particularly in Jinhae-Masan Bay and along the Busan and Geoje coasts [7,9]. Most studies of HABs in these regions have focused on understanding the population dynamics of *A. tamarense* in spring. However, little is known about toxin accumulation in filter feeder species associated with PSTs.

Ichimi et al. [10] reported that, among bivalves, mussels do not selectively accumulate particular toxins because there is no major difference in the toxin proportions in mussels and *Alexandrium*. Marsden and Shumway [11] demonstrated that mussels do not show an adverse reaction when they feed on PST-producing dinoflagellates including *Alexandrium* species. Bricelj et al. [12] and Li et al. [13] reported that mussels of the genus *Mytilus* typically become toxic earlier because they have higher toxin uptake rates than other bivalves. For this reason, members of the genus *Mytilus* are considered to potentially be useful indicator organisms for assessing toxin accumulation in relation to filter feeders and PST-producing micro-organisms during dinoflagellate bloom periods under natural conditions. Thus, to better understand the mechanisms of PST accumulation in the mussels, we investigated variation in PST composition and quantity in mussels (the genus *Mytilus*) in accordance with dynamics of *A. catenella* using natural samples which were collected from the Geoje coast (southeastern coast of Korea) for the two years (2017 and 2018). Based on previous findings [14], PST production of *Alexandrium* species is clearly varied depending on environmental conditions, and we also investigated environmental factors affecting the dynamics of PST-producing dinoflagellates during the study period.

## 2. Results

### 2.1. Wind and Nakdong River Discharges Affecting the Geoje Coast

**Figure 1:**
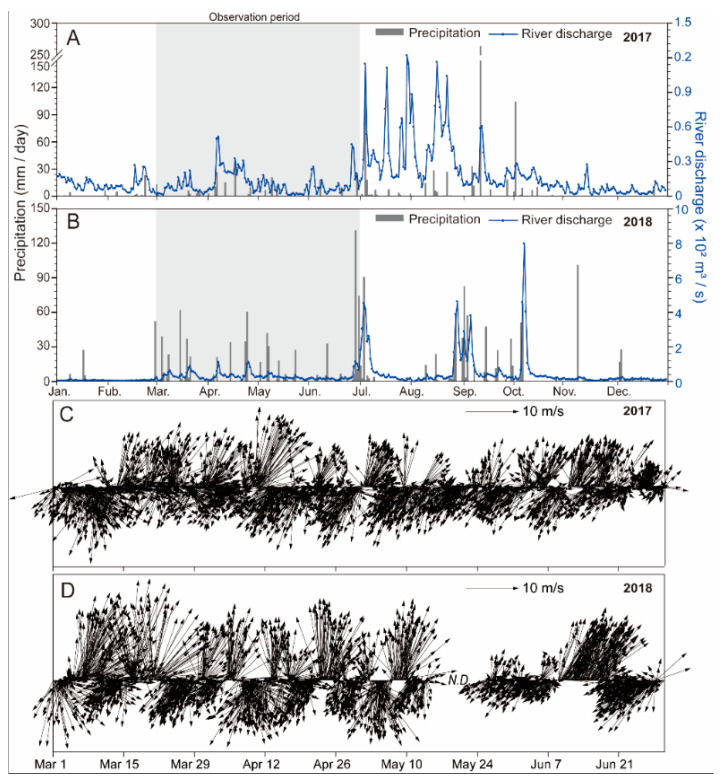
Daily precipitation and discharges from the Nakdong River in 2017 (**A**) and 2018 (**B**). The sampling periods are shaded. Time series variations in wind direction and speed during the sampling periods in 2017 (**C**) and 2018 (**D**).

Daily precipitation and rainfall flux data for the Nakdong River for 2017 and 2018 are shown in Figure 1A,B, respectively.

In both years the daily discharge and precipitation followed generally similar patterns. The seasonal discharge and precipitation were relatively high during the summer monsoon season, but they were low in the winter season. During spring season, the frequency of events involving >50 mm precipitation was higher in 2018 than in 2017. As a result, moderate discharge from the Nakdong River was recorded in 2017 (on 7 April), while three moderate discharges occurred in 2018. The time series of wind gust speed and direction recorded at the Geoje ocean data buoy are shown in Figure 1C,D, respectively. Relatively high wind speeds (>10 m s^−1^) were recorded during the study period. In terms of wind direction, southerly winds dominated (approximately 70%) in 2017, while in 2018 the wind directions were predominantly southerly or northerly.

### 2.2. Physicochemical Factors along the Geoje Coast

**Figure 2:**
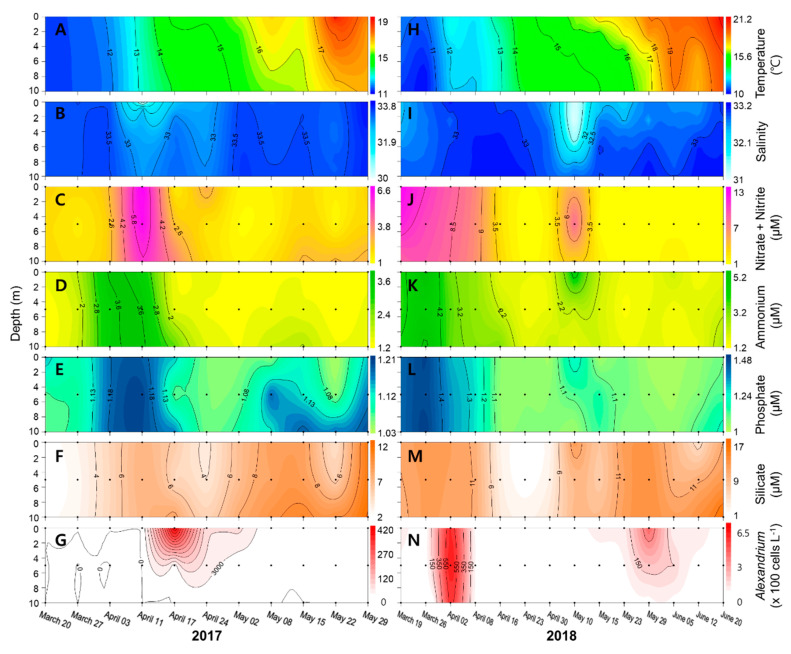
Vertical profiles of water temperature (**A**,**H**), salinity (**B**,**I**), and the concentrations of nitrate + nitrite (**C**,**J**), ammonium (**D**,**K**), phosphate (**E**,**L**), silicate (**F**,**M**), and *A. catenella* cells (**G**,**N**) from March to June 2017 (left panel) and 2018 (right panel) at Station K on the Geoje coast, Korea.

During the study periods in 2017 and 2018, the water temperature ranged from 10.3 to 21.4 °C, and gradually increased from March to June (Figure 2). Water column mixing was observed in March, and water column stratification gradually developed from May in both years. There was no clear difference in salinity levels in 2017 and 2018, and its level at the surface was generally lower than at the bottom layer. The salinity declined abruptly on 11 April 2017 (approximately 29.3) and on 10 May 2018 (approximately 31.1). During the study, all nutrient concentrations (nitrite + nitrate, ammonium, phosphate, and silicate) were relatively high during the water mixing period in March, and were relatively low in the surface layer during stratification periods. Compared with other weeks, for most nutrients, relatively high concentrations were found on 11 April 2017 and 10 May 2018, corresponding to the low salinity levels.

### 2.3. Dynamics of Alexandrium Species

*Alexandrium* species in samples was identified based on sequence analysis of ribosomal RNA gene (data not shown), and *A. catenella* was responsible for blooms during the study period. The average abundance of *A. catenella* was 2.19 × 10^3^ cells L^−1^ in 2017 and 0.81 × 10^3^ cells L^−1^ in 2018. The maximum vegetative cell density of *A. catenella* was observed on 17 April 2017 and 2 April 2018. In 2017, blooms of *A. catenella* occurred in the surface layer after one week of high nutrient concentrations associated with low salinity water discharge from the Nakdong River. Subsequently, *A. catenella* abundance rapidly decreased with increasing water temperature (approximately 18 °C) on 7 May 2018, and a small population of *A. catenella* was observed in March. The first *A. catenella* concentration peak was apparent throughout the water column on 2 April, which coincided with high nutrient levels. Thereafter, from the middle of April to the end of May, the vegetative cell concentration was <10 cells L^−1^ or not detectable. The second concentration peak of *A. catenella* in the surface layer occurred after two weeks of high nutrient levels following low salinity discharge from the Nakdong River. Subsequently, the cell concentration abruptly decreased from the middle of June, when the water temperature was >20 °C in the euphotic upper layer. Principal component analysis (PCA) showed that PC1 explained 42.6% of the variance in 2017, with positive correlations for ammonium, phosphate, and precipitation, and negative correlations for water temperature and *A. catenella* (Table 1). PC2 accounted for 20.19% of the variance, with positive correlations for river discharge and nitrate + nitrite, and a strong negative correlation for salinity. In 2018, PC1 accounted for 43.6% of the variance and showed positive correlations for nitrate + nitrite, ammonium, and phosphate, and a strong negative correlation for water temperature. PC2 accounted for 20.6% of the variance, and showed positive correlations for *A. catenella* and silicate, and negative correlations with wind speed. Over the two years of the study, PC1 accounted for 44.2% of the variance and showed a strong positive correlation for nitrate + nitrite, ammonium, phosphate, and silicate, implying that these nutrients in the water column may have come from similar sources (river discharge and water mixing from bottom layers).

### 2.4. PSTs in Mussels

**Figure 3:**
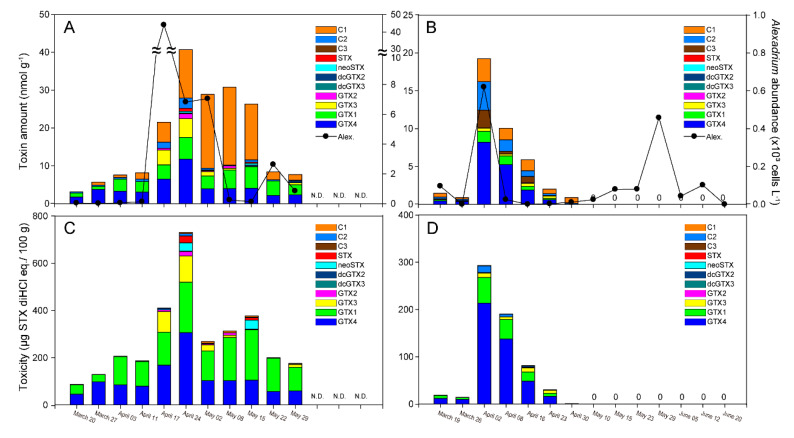
Amount (**A**,**B**) and toxicity (**C**,**D**) of paralytic shellfish toxins (PSTs) in *M. galloprovincialis* along the Geoje coast, southern Korea in 2017 (**A**,**C**) and 2018 (**B**,**D**). The black dotted circles indicate the *Alexandrium* populations in the surface layer.

The profiles of PSTs in mussels collected in the field are shown in Figure 3. In 2017, the toxicityin mussels in March was 86.6–129.7 µg saxitoxin (STX) diHCl equivalents 100 g^−1^, and gradually increased toward April. After a peak on 24 April (731 μg STX diHCl equivalents 100 g^−1^), the toxin level gradually decreased to May. From March to May, the toxin concentration in mussels exceeded 80 μg STX diHCl equivalents 100 g^−1^, which is the quarantine limit for consumption in Korea. The toxin composition predominantly comprised gonyautoxin (GTX)1 and GTX4, which accounted for approximately 70% of the total, with the toxins C3, saxitoxin (STX), neoSTX, dicarbamoylgonyautoxin (dcGTX)2, dcGTX3, and GTX2 being minor components. In 2018, toxins were detected from March, and the concentration peaked on 2 April at 293 μg STX diHCl equivalents 100 g^−1^. Toxins in mussels were recorded until the middle of April but not thereafter. The toxicity of GTX4 and GTX1 dominated in both years. The toxicity of the *N*-sulfocarbamoyl derivatives C1 + C2 toxin were relatively low compared with the neoSTX carmabate derivatives GTX4 and GTX1. In addition, the decarbamoyl derivatives dcGTX2 and dcGTX3 were detected at low levels in natural mussels.

## 3. Discussion

### 3.1. PSTs Outbreaks and Management in Southern Korean Coastal Waters

In Korea, the mussel *M. galloprovincialis* is the second most preferred shellfish after oysters. Even though mussels are widely consumed, little attention was paid to the possibility of toxin contamination prior to the first PSP incident in 1986 [7]. Since then, PSP has been identified as a potential threat to public health and a major problem in the shellfish farming industry. A second PST outbreak occurred in 1996 on the Geoje coast [8], associated with consumption of the mussel *M. galloprovincialis* from coastal waters off Busan and Jinhae Bay. Subsequently, the Korean government has paid more attention to the potential for PST outbreaks in spring, and has early PST warning systems for the initiation of public action plans. Monitoring for shellfish toxicity is conducted in shellfish-producing areas to prevent harvesting and marketing of shellfish having a PST concentration exceeding the quarantine limit (80 μg STX diHCl equivalents 100 g^−1^). For public health and the development of marine culture industries, it is urgent to have a monitoring program and impose regulations to secure the safety of shellfish. Since 2000, information on the distribution of PST-producing species and PST outbreaks in Korean coastal waters has been provided weekly on the National Institute of Fisheries Science (NIFS) website, (http://www.nifs.go.kr/bbs?id=shellfish). Accordingly, fisherman do not harvest marine organisms including shellfish when the PST concentration exceeds 80 μg STX diHCl equivalents 100 g^−1^ in the meat or midgut gland of marine organisms, particularly shellfish and Korean sea squirts.

**Figure 4:**
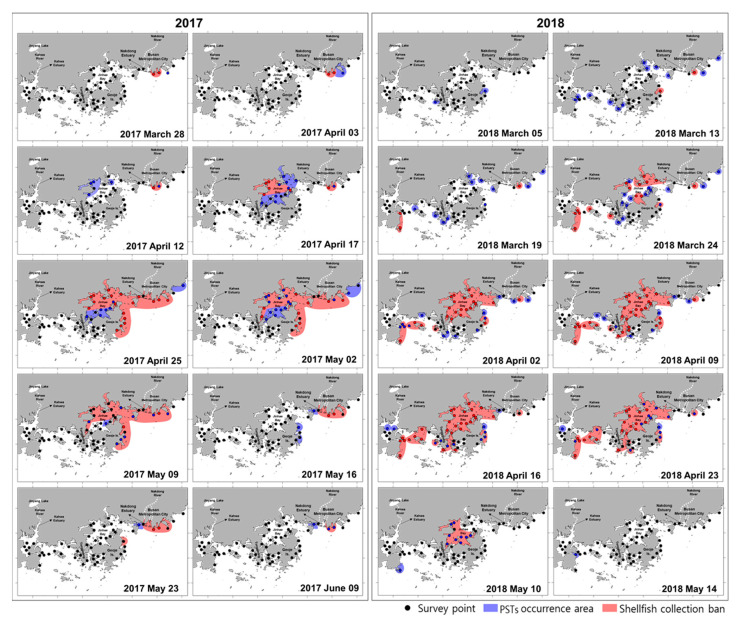
Examples of national watch service maps showing the spatial occurrence of PSTs and areas where shellfish collection has been banned in the southeast coastal area of Korea. The maps were provided by the National Institute of Fisheries Science (NIFS) and redrawn.

Information on the spatial occurrence of PSTs via the national watch service (the NIFS; http://www.nifs.go.kr/bbs?id=shellfish) has often recorded the initiation of PST occurrence in the southeastern coastal area of Korea (Figure 4).

Based on the information on PSTs from the NIFS during our study period, the PST outbreak in 2017 started along the Busan coast on 3 April (shellfish collection was banned at this time), implying that the causative *Alexandrium* sp. may have been ingested by shellfish at low population densities. On 12 April, PST outbreaks had occurred in parts of the western area of Jinhae Bay, and a shellfish collection ban was imposed for one week at this area. From 25 April to 9 May, the PST outbreak and shellfish collection ban area were greatly expanded to the Geoje and Busan coasts in addition to Jinhae Bay. From 16 May, the outbreak gradually decreased toward June, with increasing water temperature. Thus, the annual trend in PST outbreaks caused by *A. catenella* was similar to that reported by Lee et al. [15] and Kim et al. [16]. In 2018, the PST outbreak was detected on 5 March, and a shellfish collection ban was imposed (i.e., approximately one month earlier than in 2017). Thereafter, the PSTs outbreak area gradually increased to most southeast coastal areas of Korea, including Tongyeong; this was a different trend to that in 2017. Therefore, the PST outbreak patterns were different between 2017 and 2018. This difference may be related to distinct dynamic patterns of the toxin-producing dinoflagellate *A. catenella* in the two years, as noted below.

### 3.2. Dynamics of A. Catenella in the Geoje Coast

Multiple biological, oceanographic, and meteorological factors are generally important in determining the initiation, development, and decline of HAB species [6,17]. The initial introduction of viable vegetative *A. catenella* cells into the water column can play a crucial role in the development of blooms. This dinoflagellate has been shown to produce resting cysts, and is widely distributed throughout southeast Korean coastal waters [11,17]; the germination of resting cysts may have contributed to the initial presence of vegetative cells. It is widely known that water temperature plays a fundamental role in the germination of cysts, and in the growth of vegetative cells. Kim et al. [16] showed that in Korean coastal waters, *A. tamarense* (=*A. catenella*) populations increased significantly from March to May, rapidly decreased from June when the water temperature again exceeded 20 °C, then briefly reappeared in November and December. This implies that there are two peaks annually, in spring and autumn. Ichimi et al. [10] reported that an *A. tamarense* strain grew within a broad temperature range, but with an optimum of 15 °C. In the present study, *A. catenella* cells appeared when the temperature was 10–19 °C, indicating that this temperature range was suitable for *A. catenella* growth. Our field results are similar to previous reports over the past two decades by Lee et al. [15] and Kim et al. [16], suggesting that seasonal blooms of *A. catenella* mainly occur during spring in Korean coastal waters. In particular, high *A. catenella* cell concentrations were present when the nutrient concentrations were relatively high during March and April, as noted below. These findings suggest that, given its relatively narrow optimum temperature range (12–15 °C), the growth of *A. catenella* populations may be positively affected by this factor, particularly in early spring and late autumn.

The Tsushima Warm Current strongly flows into the offshore area of Geoje, and this creates a counterclockwise current. This current allows that our sampling site (the Geoje coast) is able to be largely affected by freshwater discharges from the Nakdong River during heavy rainfall periods. Baek et al. [18] demonstrated that Nakdong River discharges are associated with a negative relationship between salinity and nitrate + nitrite during winter and spring. In the present study, the salinity decreased from 33.5 to 32.5 in early April 2017 after a rainfall event, resulting in the nitrate + nitrite concentration increasing simultaneously with a decrease in salinity, and an *A. catenella* bloom occurred one week following the increased nitrate + nitrite level. Similarly, in 2018 a small *A. catenella* bloom occurred two weeks following low salinity levels and high nitrate + nitrite concentrations associated with a Nakdong River discharge on 5 May. These findings suggest that abundant nutrients supplied by freshwater input from the Nakdong River may allow the generation of blooms of *A. catenella*. In addition, based on a previous study [19], humic substances from rivers are capable of stimulating the growth of *Alexandrium* species. Therefore, an increase in humic substances due to the introduction of river discharge may also contribute to the formation of an *A. catenella* bloom.

Wind-driven surface currents have a major impact on the transport of phytoplankton blooms, and can be a key regulator of cell accumulation along coasts [20,21,22]. Based on our field observations and PST outbreak information from the NIFS, during April to May in 2017 and March to April in 2018, the population of *A. catenella* gradually expanded around Geoje Island, including Jinhae Bay. During these periods, the wind speeds were mostly >10 m s^−1^, although the wind directions varied (Figure 1). Strong winds and the low surface water temperature (because of the sinking of high-density water) caused physical acceleration of mixing of the entire water column in March and April, resulting in nutrient loading to the nearby euphotic layer from bottom layers, particularly in shallow coastal areas. The nutrient supply in the water column may have played an important role in the proliferation of *A. catenella* populations. In addition, horizontal transport by local currents (tidal effects and wind-driven surface currents) may have contributed to the movement of *A. catenella* around Geoje Island, which was a PST outbreak area, based on the NIFS information. In both 2017 and 2018, the PST outbreaks initially occurred along the Bussan coast near the Nakdong River, and in Jinhae Bay and the Geoje coast, implying that PST-producing *A. catenella* populations may have developed in these areas in spring. As a result, blooms of *A. catenella* along the Geoje coast may have been enhanced by tidal currents and wind-driven surface currents, in addition to population proliferation under optimum temperature conditions. Pettersson and Pozdnyakov [23] reported that topography and shore geometry can significantly influence cell accumulation and HAB dynamics. In addition, Anderson et al. [17] demonstrated that physical/biological coupling factors have a critical role in *Alexandrium* bloom dynamics, including determining population accumulation and growth, and the dispersal of cells in embayments and coastal waters. Thus, our results indicate that physical forcing by wind and local tidal currents is critical in controlling the horizontal and vertical distribution of *A. catenella* populations, particularly in mixing periods during winter and early spring. More detailed modeling is needed to clarify the relationship of *A. catenella* dynamics to physical forcing.

### 3.3. PSTs in M. galloprovincialis and A. catenella

The toxin levels in PST-contaminated shellfish tend to vary among seasons because there are differences in the optimum water temperature for PST-producing species. Under natural conditions in temperate regions, the occurrence PSTs tends to be high in relation to *Alexandrium* blooms when the water temperature is approximately 15–20 °C in spring [15,16,24,25]. However, PST outbreaks have occurred in subtropical and topical areas of the Pacific Ocean, including during December to February in Papua New Guinea [26] and in April in the Philippines [27]; these have been associated with blooms of the toxic dinoflagellate *Pyrodinium bahamense*. In colder areas including Alaska, some marine bivalve mollusks maintain dangerous toxin levels throughout the year [28]. In culture experiments, Han et al. [29] reported that under N-enriched culture conditions, *A. pacificum* had a a more diverse toxin profile than in P-enriched culture. In contrast, under P limitation conditions, axenic *A. pacificum* was shown to have enhanced cellular toxin production. Oh et al. [24] showed that *A. tamiyavanichii* strains isolated from temperate waters of the Seto Inland Sea (Japan) contained C2 and GTX4 as the major toxins, and C3+C4, GTX2, GTX 5, neoSTX, and STX as minor components. Similar to the findings of Han et al. [29], the *A. pacificum* cellular toxins comprised C2 > C1 > GTX1 > GTX3, although the toxin components depended on the nutrient conditions and dilution rates. This implies that there is considerable variation as a result of nutrient stress or differences in growth conditions. In the present study, when the *A. catenella* bloom occurred on 24 April 2017, the *A. catenella* cells contained C1, GTX1, and GTX2 as the major toxins (Table 2). In contrast, based on the toxin peak on 24 April, the mussel *M. galloprovincialis* contained high levels of toxins in the order of GTX4 > GTX1 > GTX3 > STX > GTX2 > neoSTX > dcGTX2 > dc GTX3. Although high proportions of C1 + C2, and GTX1+2 were detected in *A. catenella,* these toxins were at low levels in mussels. This implies that N-sulfocarbamoyl toxins (C1 and C2) biosynthesized in *A. catenella* cells might be converted to highly toxic carbamate derivatives (GTX1-4, STX, and neoSTX) and decarbamoyl derivatives (dcGTX2,3) after being ingested by mussels. Interestingly, PST compositions in the mussels were clearly varied during the study period. However, it is unclear which factor (e.g., genetic difference among *Alexandrium* populations or distinct metabolism depending on different environmental conditions) enables the induction of variation in the composition of PSTs in the mussels, and further study is necessary to resolve this.

Cembella [30] reported that the toxin content of *A. catenella* was correlated with low temperatures, resulting in high levels of PSTs per cell. Ichimi et al. [10] also found that there was an inverse relationship between toxin content and growth rate, with increases in cellular toxicity related to decreased growth rates at lower temperatures. In the present study, an *A. catenella* bloom occurred at 12–15 °C, as noted above, and the PST content of mussels was high during March and April, when the temperature was approximately 15 °C. During these periods, the toxin levels exceeded safe levels for human consumption (80 μg STX diHCl equivalents 100 g^−1^), and this corresponded approximately with bans by the NIFS on shellfish collection. Thus, the PST content of mussels may be annually influenced by the *A. catenella* biomass, which may be enhanced by low temperature conditions. Our results indicate that temperature is important for *A. catenella* growth, but is also a determining factor in the concentration of toxin in mussels.

## 4. Conclusions

The major findings of our study are that—(i) variation in environmental conditions (e.g., inorganic nutrients) due to introduction of river discharge and wind-driven currents which enables the accumulation of *A. catenella* cells contributed to the formation of *Alexandrium* blooms in the Geoje coast; (ii) PST contents in the mussel *M. galloprovincialis* were associated with abundance of *A. catenella*; and (iii) PST compositions in the mussel were clearly different from those in *A. catenella* cells. These findings can provide a deeper understanding of the mechanism of toxin accumulation in the mussels due to PST-producing dinoflagellates in the Geoje coast.

## 5. Materials and Methods

### 5.1. Study Area

The Geoje coast is centrally located in southern Korean coastal waters. Based on data obtained over the past 30 years, *A. catenella* blooms generally occur along the Geoje coast and in nearby Jinhae-Masan Bay. Since the first record of a bloom in the southeast coastal waters of Korea in 1986 [7], regular spring outbreaks of PSTs have been reported (NIFS). The Geoje coast is affected by ocean currents including the Jeju Warm Current (JWC) and the Tsushima Warm Current (TWC). In addition to the introduction of offshore water, the study site was significantly affected by coastal water currents of the Nakdong River and Jinhae-Masan Bay by tidal effects. The annual rainfall in this area is substantial (1200–1600 mm). The Nakdong River is the second largest river in South Korea, with 20% of its discharge occurring during the dry season (fall and winter) and 60–70% occurring during the summer monsoon season. This indicates that there is a large seasonal difference in the abiotic environmental conditions in the Geoje coastal region, which is open to the outer sea (resulting in intrusion of warm oceanic waters) and is affected by freshwater from the Nakdong River to the northeast.

### 5.2. Field Water Sampling and Analyses

**Figure 5:**
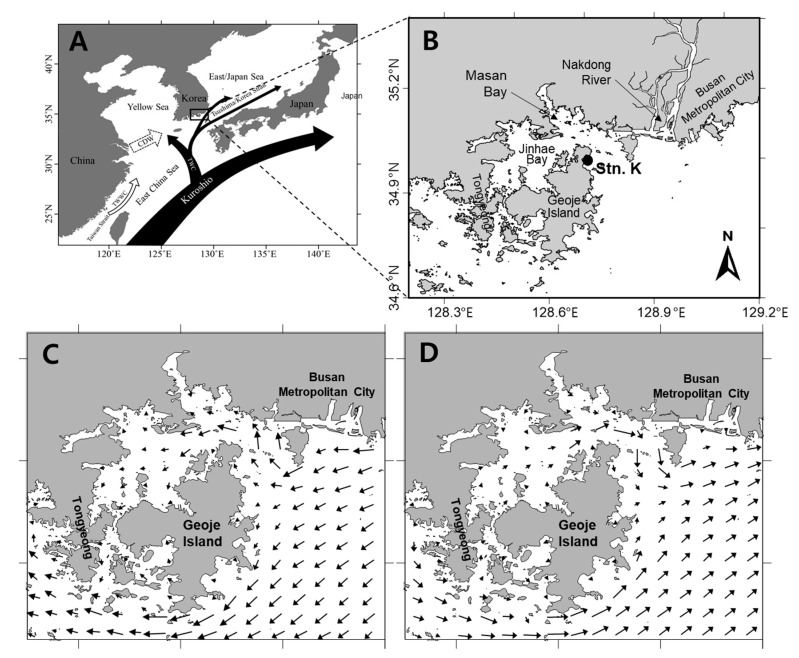
Geography and current system around Korean Peninsula (**A**). Location of the study area (Station K) on the southern coast, Korea (**B**). Direction of tidal flood (**C**) and ebb (**D**) currents in Jinhae Bay and on the Geoje coast.

To enable monitoring of the population dynamics of *A*. *catenella*, a study site (Station K: 128°42′23″ E, 34°59′36″ N) was established in the eastern coastal waters of Geoje Island (Figure 5). The sampling site is in nearshore waters of approximately 12 m depth. During spring (March to June) in 2017 and 2018, water samples were collected weekly at 0, 5, and 10 m depth using a 5 L PVC Niskin sampler. For quantitative analysis of *Alexandrium* cells, 2 L water samples collected at each water depth were stored immediately in polyethylene bottles and fixed with Lugol’s solution (final concentration: 2%). The fixed water samples were left to facilitate particle settling, then reduced to approximately 5–50 mL by decanting the supernatant. The concentrated subsamples were loaded onto a Sedgewick–Rafter counting chamber after gentle mixing, and cells of the *Alexandrium* cells were counted using a light microscope (Axioskop 2, Carl Zeiss; Jena, Germany) at 200–400× magnification. For species identification, detail morphology of *Alexandrium* cells was observed using a scanning electron microscope (JSM 7600F, JEOL; Tokyo, Japan) and molecular analysis of the cells was also conducted. For nutrient measurements, water samples were immediately filtered through a 47-mm diameter GF/F filter (Whatman). The filtrates were placed in acid-cleaned polyethylene bottles, fixed by the addition of HgCl_2_, and stored in the dark at −20 °C until laboratory analysis. The concentrations of inorganic nutrients (ammonia, nitrate, nitrite, phosphate, and silicate) were measured using a flow injection autoanalyzer (QuikChem 8000; Lachat Instruments, Loveland, CO, USA), which was calibrated using standard brine solutions (RMNS, KANSO Technos Co., Ltd.; Osaka, Japan).

### 5.3. Mussel and Plankton Sampling for Toxin Analysis

Mussel samples were collected weekly from March to June in 2017 and 2018 from a shellfish farm near the water sampling station. The mussels were attached to long ropes suspended at approximately 2 m depth at the farm. Ten mussels of similar size were sampled in triplicate in 2017 and five were sampled in triplicate in 2018. The mussels were shucked and dissected to remove the flesh from the shell. The flesh was freeze-dried (Martin Christ; Gamma 1-16 LSCplus), homogenized, and stored until analyzed for toxin content. Following this, a 4 L water sample was collected at the surface on 24 April 2017 when the *Alexandrium* bloom occurred, and filtered through GF/F. The filtrate was stored at −20 °C until toxin analysis.

### 5.4. PSTs Analysis

**Figure 6:**
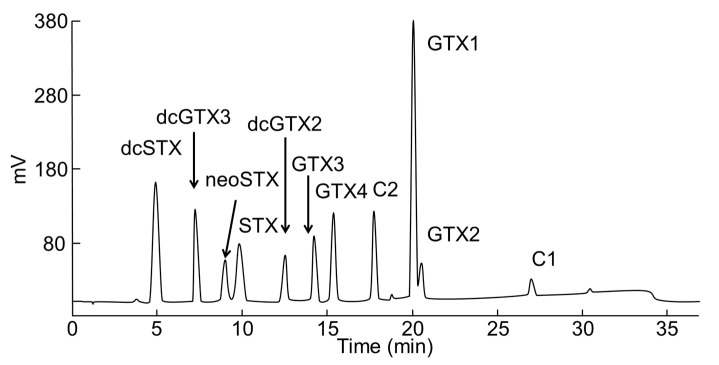
A typical chromatogram of the PST standards.

PSTs were analyzed using HPLC (High-performance liquid chromatography) with postcolumn derivatization. [31,32]. HPLC analysis was performed using an Alliance 2690 Separations Module (Waters) fitted with porous graphitic carbon Hypercarb^®^ column (4.6 mm i.d. × 100 mm length; 5 µm Thermo Fisher Scientific) at 25 °C. The separation of toxins was performed using two mobile phases—(A) 0.075% (*v*/*v*) TFA (Trifluoroacetic Acid) in water; (B) 0.025% (*v*/*v*) TFA in 50% (*v*/*v*) acetonitrile: water. The flow rate was set at 0.8 mL/min. Initial conditions were 4% B, followed by a linear gradient from 4% B to 25% B over 30 min, then returned to 4% B at 30.01 min, and held and re-equilibrated for over 7 min until the next injection. The eluate from the column was mixed continuously with 50 mM periodic acid and 0.2 M KOH containing 1 M ammonium formate and 50% formamide, and heated at 65 °C. The formation of fluorophores was monitored at 392 nm using 336 nm excitation. Reference materials of C1, C2, C3, GTX1-4, and dcGTX2,3 were provided by the Japan Fisheries Research and Education Agency, and neoSTX, dcSTX, and STX purified from the toxic crab *Zosimus aeneus* [33] were used as external standards to identify/quantify individual analogues (Figure 6). The toxicity of each PST was calculated with the recommended toxicity equivalency factors (TEFs) for the STX group [34].

### 5.5. Hydrological and Meteorological Environment Data

Water temperature and salinity were recorded at each sampling depth of the sampling site using CTD (Ocean Seven 319, Idronaut Co.; Brugherio, Italy). Meteorological data (daily precipitation and discharges from the Nakdong River, and wind speed) were accessed through the Korea Meteorological Administration (KMA; http://web.kma.go.kr). Wind speed was measured from coastal weather stations (34°46′00″ N, 128°54′00″ E).

### 5.6. Statistical Analysis

The field data on biotic and abiotic factors from the inshore and offshore stations were analyzed and compared using a t-test. Differences were considered significant at the *p* < 0.05 level. One-way ANOVA followed by Tukey’s test was used to detect significant differences in water temperature and salinity over the past five years (2012–2016) along the Tongyeong coast. All statistical analyses were performed using SPSS version 17.0 (SPSS Inc.; Chicago, IL, USA). 

## Figures and Tables

**Table 1:** Factor loadings for two principal component correlations between *A. catenella* populations and environmental variables. Bold values indicate statistically significant in the level of <0.05.

	Total Survey		2017		2018	
	PC1	PC2	PC1	PC2	PC1	PC2
Temperature	−0.494	0.076	−**0.802**	−0.202	−**0.862**	0.409
Salinity	−0.133	−**0.816**	−0.197	−**0.941**	0.091	−0.179
NO_x_	**0.882**	0.210	0.421	**0.801**	**0.982**	0.029
NH_4_	**0.811**	0.370	**0.779**	0.362	**0.825**	0.094
PO_4_	**0.938**	0.096	**0.655**	0.305	**0.961**	0.073
SiO_2_	**0.682**	0.155	−0.204	−0.078	0.460	**0.761**
Wind speed	0.220	0.240	0.285	0.125	0.475	−**0.666**
R. discharge	0.149	**0.766**	−0.045	**0.864**	0.368	−0.571
Precipitation	0.329	**0.660**	**0.647**	0.328	0.337	−0.255
*Alexandrium*	0.005	−0.459	−**0.883**	0.230	0.034	**0.685**
Eigenvalue	4.42	1.46	4.26	2.02	4.36	2.06
Variability (%)	44.24	14.60	42.63	20.19	43.65	20.58
Cumulative (%)	44.24	58.84	42.63	62.82	43.65	64.23

**Table 2:** Toxin content (fmol cell^−1^) and toxicity (μg STX diHCl equivalents cell^−1^) of a surface water sample collected at the blooming time (24 April 2017) of *A. catenella*.

	Toxin Content		Toxicity	
	fmol cell^−1^	Ratio (%)	μg STX diHCl equivalents cell^−1^	Ratio (%)
C2	0.33	1.54	0.0012	1.17
GTX2	2.90	13.37	0.0432	41.09
GTX1	1.46	6.71	0.0543	51.71
C1	17.01	78.37	0.0063	6.03
**Total**	21.70	100	0.11	100
